# Genome annotations for the ascomycete fungi *Trichoderma harzianum*, *Trichoderma aggressivum*, and *Purpureocillium lilacinum*

**DOI:** 10.1128/mra.01153-23

**Published:** 2024-02-22

**Authors:** Erik P. W. Beijen, Robin A. Ohm

**Affiliations:** 1 Department of Biology, Microbiology, Faculty of Science, Utrecht University, Utrecht, the Netherlands; University of Guelph, Canada

**Keywords:** fungal genomics, ascomycetes, mycoparasites

## Abstract

We sequenced and annotated the genomes of the ascomycete fungi *Trichoderma harzianum*, *Trichoderma aggressivum f. europaeum*, and *Purpureocillium lilacinum*. Moreover, we developed a website to allow users to interactively analyze the assemblies, gene predictions, and functional annotations of these species and 70+ previously sequenced fungi.

## ANNOUNCEMENT

*Trichoderma harzianum* and *Trichoderma aggressivum* are mycoparasites that can cause green mold disease on mushroom-forming fungi, including the white button mushroom *Agaricus bisporus* (1). Phylogenetically, they are members of the Harzianum/Virens clade of the *Trichoderma* species complex (2). The species *T. aggressivum* encompasses the biotypes *aggressivum* and *europaeum*, which originate from North America and Europe, respectively (3). The ascomycete *P. lilacinum* is a ubiquitous saprotroph, found in soil, dead organic matter, insects, and air (4). We serendipitously discovered that it can inhibit growth of the mushroom-forming fungus *Schizophyllum commune*.

*T. harzianum* CBS 354.33, originally isolated from soil, and *T. aggressivum* CBS 100526, originally isolated from mushroom compost, were obtained from the Westerdijk Institute. *P. lilacinum* was isolated from the air and deposited in the Westerdijk collection with accession number CBS 150709. A spore suspension was incubated for 3 days at 30°C in liquid culture in *Schizophyllum commune* minimal medium (5). Genomic DNA was extracted and purified from lysed cells using the MagMAX Plant DNA Isolation Kit (Thermo Scientific, USA) and sonicated using a Bioruptor (Diagenode, Belgium) for 10 minutes, with a 30-/90-second on/off cycle on low power and the temperature in the water bath at 4°C, resulting in fragments of approximately 700 bp. The NEBNext Ultra II DNA Library Prep Kit for Illumina (New England Biolabs) was used to generate Illumina libraries. These libraries were sequenced by the Utrecht Sequencing Facility (Utrecht; The Netherlands; https://useq.nl) using an Illumina NextSeq2000 sequencer with a 2 × 100 bp protocol. The reads were trimmed on both ends to a quality score of 15 using BBMap version 37.88 (https://sourceforge.net/projects/bbmap/) and assembled with SPAdes version 3.15.5 (6) with kmer lengths 21, 33, 55, 77, and 99 and the parameter “--careful.” Scaffolds shorter than 1,000 bp were removed from the assembly. Genes were predicted with AUGUSTUS version 3.3.2 (7) using the provided parameter set of the related ascomycete *Aspergillus nidulans* and the settings “--genemodel=complete” and “--noInframeStop=true.” The predicted proteins were functionally annotated as previously described (8). Repetitive sequences were identified with RepeatModeler version 2.0.3 (9).

A website was created with a portal for each genome and for all 70+ genomes that were previously sequenced and published by us (https://fungalgenomics.science.uu.nl/). The genome portals allow users to interactively analyze the assembly, gene predictions, and functional annotations. This includes a JBrowse genome browser (10) with Apollo plugin to allow the manual curation of gene models (11), a Blast function to search for sequence similarity in the assembly and gene predictions (12), and full access to the functional annotations of the proteins.

The sizes of the genome assembly were 38.51, 38.95, and 40.62 Mbp for *T. harzianum*, *T. aggressivum*, and *P. lilacinum*, respectively, and BUSCO completeness scores were 99.66% for both *Trichoderma* spp. and 98.62% for *P. lilacinum*, indicating a high quality of assembly and gene prediction for all three species (Table 1). These genome annotations and the genome portals will be a valuable resource to study these three competitors of mushroom-forming fungi.

**TABLE 1:** Statistics of the three genome annotations

	*Trichoderma harzianum*	*Trichoderma aggressivum f. europaeum*	*Purpureocillium lilacinum*
Strain	CBS 354.33	CBS 100526	CBS 150709
Sequencing read pairs	23,531,919	18,241,525	19,347,672
Version of assembly and annotations	Trihar35433_1	Triagg1	Purlil1
Assembly			
Sequences in assembly	171	318	928
Coverage	122×	93×	100×
Total assembly length (Mbp)	38.51	38.95	40.62
Assembly GC^*a*^ content (%)	49.3	49.06	58.33
Assembly gaps (%)	0	0.01	0.02
L50 number (#)	6	20	25
N50 length (bp)	2,296,050	579,860	470,782
Repetitive content (%)	4.23	1.9	3.99
Predicted genes			
Genes	11,326	11,015	14,434
Gene length (median)	1,422	1,431	1,489
Transcript length (median)	1,281	1,281	1,296
Exon length (median)	297	298	288
CDS length (median)	1,278	1,278	1,293
Protein length (median)	426	426	431
Spliced genes (total, %)	8,366 (73.87%)	8,160 (74.08%)	10,853 (75.19%)
Exons per gene (median)	2	2	3
Intron length (median)	65	66	65
Introns per spliced gene (median)	2	2	2
Gene density (genes/Mbp)	294.09	282.79	355.36
Coding content of assembly (bp, %)	17,783,979 (46.18%)	17,306,514 (44.43%)	23,597,817 (58.1%)
Functional annotations			
Unique PFAM domains	4,125	4,118	4,129
Genes with PFAM domain (total, %)	8,661 (76.47%)	8,226 (74.68%)	8,386 (58.1%)
Genes with GO annotation (total, %)	5,414 (47.8%)	5,142 (46.68%)	5,360 (37.13%)
Genes with secretion signal (total, %)	1,053 (9.3%)	970 (8.81%)	1,259 (8.72%)
Genes annotated as small secreted protein (total, %)	911 (8.04%)	991 (9.00%)	1,146 (7.94%)
Genes with transmembrane domain (total, %)	2,141 (18.9%)	2,063 (18.73%)	2,543 (17.62%)
Genes annotated as transcription factor (total, %)	487 (4.3%)	437 (3.97%)	497 (3.44%)
Genes annotated as protease (total, %)	477 (4.21%)	438 (3.98%)	474 (3.28%)
Genes annotated as CAZyme (total, %)	456 (4.03%)	430 (3.9%)	454 (3.15%)
Secondary metabolism gene cluster	54 (0.48%)	58 (0.53%)	32 (0.22%)
BUSCO2 completeness (%)	99.66% (single copy: 99.31%, duplicated: 0.34%)	99.66% (single copy: 99.66%, duplicated: 0.0%)	98.62% (single copy: 97.93%, duplicated: 0.69%)

^*a*^ guanine-cytosine.

## Data Availability

All data are available under NCBI BioProject PRJNA1034581. The raw sequence reads of *T. aggressivum*, *T. harzianum*, and *P. lilacinum* are deposited in NCBI Sequence Read Archive (SRA) under accession numbers SRR26638389, SRR26638390, and SRR26638391, respectively. The genome assemblies and gene predictions are deposited in NCBI GenBank under accession numbers JAWRVG000000000, JAWRVH000000000, and JAWRVI000000000, respectively. The genome annotations can also be accessed at https://fungalgenomics.science.uu.nl.
